# Processing and preservation technologies to enhance indigenous food sovereignty, nutrition security and health equity in North America

**DOI:** 10.3389/fnut.2024.1395962

**Published:** 2024-06-19

**Authors:** Danielle Heaney, Olga I. Padilla-Zakour, Chang Chen

**Affiliations:** Department of Food Science, Cornell AgriTech, Cornell University, Geneva, NY, United States

**Keywords:** indigenous food sovereignty, nutrition security, health equity, food processing, food safety, food quality

## Abstract

Indigenous foods are carriers of traditional native North American food culture and living philosophy. They are featured by the wide varieties in fresh and processed forms, richness in nutrition, flavor, health benefits and diversity in origins, but are usually misunderstood or underrepresented in the modern food systems. Conventional processing and cooking methods are sometimes labor-intensive, less efficient and lack science-based guidelines to prevent unseen safety risks and food loss. Global and regional climate change have caused additional challenges to conventional cooking/processing, and increased native communities’ reliance on externally produced foods, which have resulted in increasing nutritional unbalance and prevalence of diet-related health issues. Current and emerging technologies, such as storage and packaging, drying, safety processing, canning, pickling, and fermentation, which treat foods under optimized conditions to improve the safety and extend the shelf-life, are increasingly used in current food systems. Therefore, exploring these technologies for indigenous foods offers opportunities to better preserve their nutrition, safety, and accessibility, and is critical for the sovereignty and independence of indigenous food systems, and sustainability of indigenous food culture. This mini-review focuses on identifying adoptable processing and preservation technologies for selected traditional indigenous foods in North America, summarizing education, extension, and outreach resources and discussing the current challenges and future needs critical to expanding knowledge about indigenous foods and improving food sovereignty, nutrition security, and health equity.

## Introduction

‘Indigenous Food Sovereignty’ is a concept drawing increasing attention (1), which states native people have ‘the right to produce healthy and culturally appropriate food through ecologically sound and sustainable methods’ (2). However, contemporary diets and increased reliance on externally produced foods have threatened the sovereignty and sustainability of native food systems and culture (3). Over-consumption of hyper-processed, calorie-dense, convenient foods has led to nutritional imbalances and increased diet-related health challenges, particularly among indigenous populations in the North America (4). Prevalence of type-II diabetes and cardiovascular diseases among indigenous populations were reported 16.5% higher, and the average life expectancy 5.2 years shorter than other races in America (5, 6). Global climate change has also resulted in challenges and uncertainty. For example, elevated ambient temperatures and more intensive rainfalls have increased emergence of crop diseases and pest damage, making food more vulnerable to decay and safety risks (7). Indigenous foods are featured by their diversity, nutritional value, and freshness, but can decay rapidly due to physical, chemical and/or microbiological changes if they are not preserved or processed appropriately (8). Processing is essential to extend the shelf life and accessibility of foods and avoid food loss. Therefore, to aid food sovereignty, food processing techniques need to be adapted to fit indigenous values.

Practicing food sovereignty in native communities is highly encouraged (9), but available information on suitable processing/preservation technologies for indigenous foods and their attributes, nutrition, safety and accessibility is limited. Instead, identifying and adopting suitable technologies for the indigenous food systems should be based on the knowledge of food attributes and their relationships with the processing/preservation conditions, while respecting the indigenous food cultures. Although numerous food processing/preservation technologies have been developed, translation and dissemination of these research outcomes to indigenous communities is limited. Addressing this will require extra efforts from institutions/agencies to identify the needs and develop suitable extension, outreach and education materials/activities, with proper and proactive communications.

**Figure 1:**
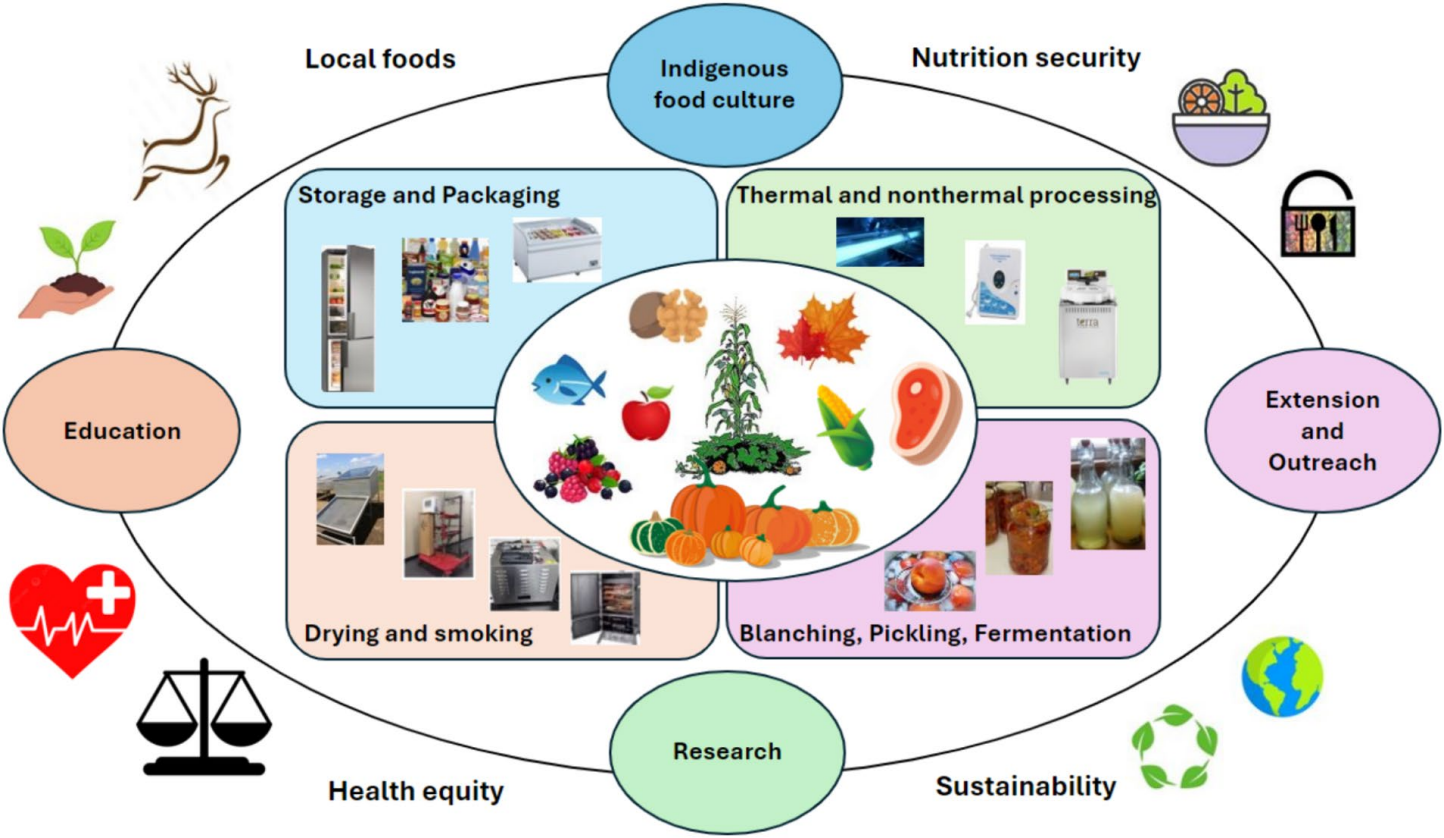
Contributions and applications of a virtualized food. Current preservation/processing technologies and indigenous food sovereignty, nutrition security and health equity.

Therefore, this mini-review aims to (1) identify and discuss emerging and accessible technologies that can help improve food safety and shelf life, preserve the nutritional value of indigenous foods, and sustain the native food culture (Figure 1); and (2) summarize the available education, extension and outreach resources. It is envisioned that this study can provide complementary information to the current reported efforts from cultural, ecological and public health perspectives (10, 11), and contribute to a more resilient indigenous food system.

### Preserving and processing technologies

Several commodity groups have been identified as important to indigenous people in North America and are summarized in Table 1. Grains, nuts, fruits, vegetable pulses, and animal proteins are among the most commonly reported (12), typically processed through refrigeration, freezing, baking, sun-drying, and roasting (21). Scoping applications of food technologies to these main commodity groups may prove a valuable resource for the creation of extension resources to improve indigenous food sovereignty while upholding their desires, needs, and values. Therefore, this paper evaluates successful applications of various food technologies to grains, nuts, fruits, vegetable pulses, and animal proteins and delineates the benefits of each technology. These technologies are summarized in Table 2.

**Table 1:** Important food commodities for native groups in Northeast America.

Commodities and prepared foods	Traditional preservation/ processing methods and products	Reference
Grains and nuts		
Corn (maize, Flint)	Grains and nuts are usually sun-dried and ambient stored, the dried materials are consumed whole, baked, boiled, ground or milled into powders, meal or paste.	(12); Niyati (13)
Black Walnut		
Chestnut		
Fruits		
Apples	Fruits are usually stored at ambient, refrigerated, or frozen temperatures. They are also sun-dried, juiced, cooked in porridge, and reconstituted into sauce. Native people also cook them with wild game meat.	(12); Niyati (13, 14)
Grape (Concord)		
Berries (strawberry, blueberry, cranberry, raspberry, blackberry)		
Pawpaw		
Vegetables		
Squash (cucumber, zucchini, pumpkin)	Vegetables are commonly stored at ambient, refrigerated, or frozen temperatures besides fresh consumption. They are also baked, salted, and sun-dried.	(15, 16)
Fiddleheads		
Potato		
Pepper (bell, chili)		
Pulses		
Black bean	Pulse crops are normally sun-dried, cooked in water, or ground into meals. Dried whole pulses or meals are usually stored at ambient temperature.	(17, 18)
Kidney bean		
Navy bean		
Lima bean		
Animal proteins		
Venison	Wild game and fish meats are salted, sun-dried into jerky, boiled, or smoked with an open flame.	(19, 20)
Bison		
Fish		
Beaver		

**Table 2:** Processing and preservation technologies that offer opportunities for indigenous foods.

Technology opportunities	Commodity groups	Infrastructure needed
Controlled condition storage: Temperature control (15) Humidity control (33) Atmosphere composition control (22)	Grains and nuts, fruits, vegetables, pulses, animal proteins	Home refrigerator and freezer, larger scale cold-storage room with controlled atmosphere with hygienic design, power (fuel and electric), instrumentation for process monitoring and control (temperature, pH, humidity, water activity, time)
Drying technologies: Indirect sun-drying (21) Hot air drying (23) Infrared drying (24) Freeze-drying (reference)	Grains and nuts, fruits, vegetables, pulses, animal proteins	Home food dehydrator, pilot-scale dryer for community use, processing space with hygienic design, power, instrumentation for process monitoring and control
Canning: Blanching (25) Thermal pasteurization (26) Acidification (27)	Pulses, fruits, vegetables, animal proteins	Home kitchen, commercial processing space with hot steam and cold water, power, instrumentation for process monitoring and control
Fermentation: Alcoholic fermentation (28) Pickling (29)	Grains, fruits, vegetables	Home kitchen, hygienic food preparation space, proper food storage space
UV treatment (34)	Grains and nuts, juices	UV lamps, at home UV food sanitizer/pasteurizer, enclosed processing space with hygienic design, power
Gas smoking (30)	Animal proteins	Gas smokers with separate cooking and smoking chambers, power, instrumentation for process monitoring and control
Packaging: Vacuum packaging (31) Canning (32)	pH and water activity-controlled foods	Proper and sanitized packaging materials (glass, metal, flexible), packaging space with hygienic design, vacuum pump, power

Food safety risks may increase if the foods are not preserved or processed properly. Pathogenic microorganisms among the biggest risks resulting in significant numbers of foodborne illnesses in the United States. Recent outbreaks in the US have traced back to eggs, meats, nuts, grains, fruits and vegetables (35). Chemical safety risks could also emerge due to inappropriate processing and cooking. For example, baking or roasting could form acrylamide, a group 2A carcinogen in bakery or meat products (36, 37). Smoking could generate carcinogenic polycyclic aromatic hydrocarbons (PAHs) and dioxin-like polychlorinated biphenyls (PCBs) in foods (38). This section also evaluates suitable processing technologies to improve the safety and shelf-life of conventional indigenous foods.

### Food storage and packaging

Proper storage methods are critical to ensure safety, prolong shelf-life and accessibility, and preserve nutrition and quality of natively consumed crops and wild games. Under uncontrolled storage conditions, quality decay and microbial growth could accelerate due to ripening and/or contamination as affected by the relative humidity (RH), temperature, and atmospheric composition (22). Freshly harvested fruits and vegetables mature and respire, losing moisture, releasing CO_2_ and undergoing biochemical reactions which alter antioxidants, proteins, carbohydrates, and color (39). Therefore, extension and outreach resources which suggest proper storage conditions and packaging can contribute significantly to the indigenous food sovereignty.

- Cold storage: Refrigeration (0–5°C) slows food respiration, reducing biochemical changes. From a quality standpoint, refrigerated storage could better maintain the firmness of tomatoes, snap beans, and blueberries (17, 40, 41), and minimize color changes (15, 42, 43). The microbial growth in other fruits, including blueberries, strawberries, grapes, and cranberries, can also be suppressed under proper storage conditions (44). Freezing (<−18°C) can further extend storage stability, particularly for meat and fish (33, 45). Suitable storage temperature and time depend highly on the food’s properties and maturity. Inappropriate refrigeration may lead to chilling injury or growth of large ice crystals in the food cells, which may counterproductively result in texture softening, discoloration, and loss of nutrition (16, 46). Indigenous communities also have the option to create and monitor suitable storage conditions at home following the recommendations of the National Center for Home Food Preservation (47).
- Packaging: Packaging also extends foods’ shelf life by providing a barrier for moisture, oxygen, and light, defending against pathogens and animals, and generating suitable gas compositions for food quality preservation. A variety of materials can be used for food packaging. Low density polyethylene bags improve cucumber shelf-life compared to aluminum foil and open-air storage (48). Metal cans and glass jars are rigid and thus suitable for processed foods like pickled vegetables or liquids (32). Vacuum sealing can significantly reduce moisture loss, protein degradation, lipid oxidation and texture toughening in wild-games before cold storage (49). More advanced storage facilities with microbial control (using ultraviolet and/or ozone generators) and modified atmosphere (e.g., low O_2_ and higher CO_2,_ ethylene absorbers) can suppress the respiration and ethylene generation rates of fresh produces and work more effectively under refrigerated conditions (50, 51).

### Drying and smoking

Drying/dehydration is one of the most traditional ways of food preservation (52). Low moisture and intermediate moisture foods have extended shelf-life. Reducing the moisture content and water activity of harvested foods can avoid pathogenic microbial growth, mycotoxin formation, decay, or spoilage (53). The sun and wind are considered as gifts by the Creator to the native people (54), and have been widely used to dry corns, beans, squashes, berries, and other fruits, vegetables and wild games in many indigenous cultures (12). However, weather conditions (>30°C, <60% humidity) can be a major concern with sun drying. The increased occurrence of intensive rainfalls in parts of North America (e.g., Northeast) during summer and early fall (when ambient temperature is >30°C) has significantly limited sun drying’s feasibility and led to food loss due to insufficient or delayed drying (55, 56). To improve the drying efficiency and better preserve foods, other drying technologies can be used.

- Indirect sun drying (ISD): Instead of heating the foods under open sun, ISD effectively collects solar radiation through a black corrugated metal sheet to heat up the air (57). A centrifugal fan is usually used to circulate the dry heated air into a drying chamber to dehydrate the foods. Compared to open sun drying, ISD enhances drying efficiency with relatively low capital and operational costs (21).
- Hot air drying (HAD): HAD relies on forced convective heat/mass transfer from electrically heated air to accelerate the drying process and is suitable for solid foods drying (23). HAD efficiency is independent of weather conditions, representing an affordable option for indigenous communities.
- Infrared (IR)-assisted drying: IR is part of the electromagnetic spectrum and the main ‘heating source’ in the sunlight. IR drying relies on radiative heat transfer to heat up the water rapidly and selectively within foods (58). Such characteristics make IR drying more intensive than convective drying, and thus suitable for surface pre-drying or thin object drying (24). IR drying has also been used to assist HAD for more efficient processing (59).
- Freeze-drying: Freeze-drying happens under vacuum and at low temperatures, where water evaporates through sublimation from pre-frozen foods. For heat sensitive foods and high-quality products, freeze-drying is the best option, but it is expensive and slow. Newer units for small-scale and household use are opening applications (60).
- Smoking: Smoking is a traditional method indigenous people use to preserve meat products like bison, fish, and venison (19). Besides the safety risks of PAHs and PCBs formation, the non-uniform heating during open fire or direct flame smoking may result in hot and cold spots in the foods, which may lead to burning, acrylamide formation, and inadequate pasteurization (61). To address these concerns, gas smokers can be used. In gas smokers, the cooking and smoking chambers are separated; smoke is delivered via convection to improve the uniformity, and foods are placed on metal trays to avoid oil dripping onto charcoals, which can significantly decrease the PAH emission (30).

It should be noted that high temperature and long drying time may cause discoloration of foods, texture shrinkage and hardening, poor organoleptic qualities, and loss of nutrients (62, 63). Therefore, it is critical to select suitable drying conditions based on the characteristics of the indigenous foods.

### Thermal and nonthermal processing

- Thermal processing: Hot water treatment is an effective and accessible method at both home and production scales to pasteurize foods. Hot water treatment at 65°C for 20 min can kill or inactivate most vegetative cells of pathogenic microorganisms, such as *Shigella, Salmonella*, and enterotoxigenic *E*. *coli* (26). Retort technologies for canned foods are available at multiple scales to achieve effective pasteurization or sterilization. Canning at high temperatures (~121°C) and anaerobic conditions are used to extend shelf-life of low acid foods. However, incorrect practices such as under processing or time–temperature abuse can lead to serious consequences, like botulism. High temperatures during canning can lead to undesirable changes like losses of vitamins (64), β-elimination reactions leading to softening especially under high pH (65), and loss of Mg^2+^ from chlorophyll in green foods, converting it to the yellow-brown pheophytin compound (66). Adjustments should be considered for improved processing of indigenous foods, such as addition of sodium carbonate to blanching water to improve color and monitoring the time–temperature conditions depending on the product to achieve commercial sterility (25). Besides for drying purpose, IR heating can be used as a dry-pasteurization technology. IR heating at 120°C then holding at 90°C for 10–15 min reduced the *Pediococcus* level on almonds by more than 5-log (67). IR heating of shelled corn at 3.24 kW/m^2^ intensity for 150 s resulted in more than 5.9 Log CFU/g reductions of *Aspergillus flavus* (68).
- Nonthermal processing: For fresh produce with heat-sensitive nutrients, nonthermal technologies can be used to disinfect the food surfaces and preserve quality (69). For example, ultraviolet treatment can be used to effectively reduce the levels of *E. coli* ATCC 11775 on lettuce, grape tomatoes and carrots (34), as well as fruit juice while maintaining desirable nutritional and sensory properties (70). Other novel nonthermal processing technologies (such as high-pressure processing, pulsed electric field, cold plasma, ionized irradiation, etc.) and hurdle technologies are used in food industries, but may not suit indigenous communities in the current stage due to expensive, specialized infrastructure requirements. Fermentation and acidification are established and accessible technologies.

### Blanching, pickling and fermentation

For long term storage of seasonal native crops with poor stability, blanching, pickling, and fermentation are useful preservation methods to ensure retention of texture, color, flavor attributes, and availability, strengthening food security. Recommendations based on particular product conditions like pH and salt level could improve both the safety and quality of indigenous foods.

- Blanching: As a common pretreatment to canning and pickling, blanching involves immersing foods in hot water or steam, then quickly cooling. Blanching elongates shelf-life by altering cell permeability and deactivating the enzymes responsible for the biochemical reactions during food decay. It can also improve the efficiency of subsequent processes like drying, freezing, or canning (18, 71). Deactivating peroxidase and lipoxygenase (90°C-100°C) can mitigate browning, rancidification and off-flavors (64); activating pectin methylesterase (50°C-70°C) in vegetables leads to texture firming through pectin de-esterification when followed by calcium gelation (72); deactivating polygalacturonase (around 80°C) prevents depolymerization of pectic acids, which results in softening. Blanching can also brighten food color, as it allows permeating water to remove intercellular gas for better light reflection (25).
- Pickling and Fermenting: Pickling and fermentation preserve fresh flavors and nutrients and add unique flavors and/or probiotics (29). During fermentation, natural or added microbes metabolize sugars in the food, producing ethanol (alcoholic beverages), CO_2_, and lactic acid which decreases the pH below 4.6 and creates an environment that is safe from most pathogens. During pickling, acetic acid and/or salt are added to achieve a pH below 4.6 without the assistance of microorganisms, to prevent botulism. A heat treatment or pasteurization step at temperatures between 70 and 90°C can be used to ensure safety under anaerobic conditions. Fermentation of meats, fish, and vegetables have existed as a part of the traditional indigenous diet (73, 74). Frybread, a fermented wheat bread, is commonly consumed since the introduction of wheat to North America (75). Unsafe pickling and fermenting practices can lead to botulism (73), thus the pH, redox potential, water activity, and storage temperature need to be controlled carefully (29).

Introduction of portable and accessible instrumentation to monitor food processes can improve food safety and quality for food sovereignty of indigenous groups. This includes pH meters, pH test strips, water activity meters, and thermometers capable of measuring internal food temperature, and monitoring fermentation vessels.

## Extension and education resources

Education and extension materials/activities are critical to disseminate the optimal technologies and food knowledge to indigenous communities for the preservation of food quality and safety. Currently available resources offered by different institutions and agencies are summarized:

- The USDA Indigenous Food Sovereignty Initiative, in partnership with tribal-serving organizations, seeks to promote traditional indigenous foods and agriculture, targeting foods that meet dietary needs. It includes educational series for children, cooking videos, and guides on foraging and harvesting indigenous plants (76).
- The Federally-Recognized Tribes Extension Program (FRTEP) supports federally recognized Indian Reservations and Tribal jurisdictions by leveraging government resources with tribal leadership through programs centered on youth education, traditional agriculture, sustainability, and community development, in collaboration with land-grant universities (77).
- The Native American Food Sovereignty Alliance has programs to help advocate and support food sovereignty, including the indigenous Seed Keepers Network that provides mentorship to famers and gardeners to reclaim and produce quality seeds for traditional indigenous foods; the Native Food & Culinary program that intents to reconnect indigenous groups with traditional diets; and the food sovereignty events (78).
- The Indigenous Food Wellness Circle program, focused on the indigenous medicine wheel, offers training courses, educational videos and resources on native nutrition, food safety, cooking demonstrations, and food preservation (79).
- The First Nations Development Institute created the Nourishing Native Foods & Health program to support tribes and native communities to establish sustainable food systems. The Native Farm to School project offers educational resources that integrate traditional foods and practices into school curricula, promoting health, self-reliance, and sustainability (80).
- The Woodland Indian Educational Programs provide guidelines for sun drying, smoking foods, and storing foods (81).
- The North American Traditional Indigenous Food Systems established the Indigenous Food Lab, a professional kitchen and training center that covers indigenous food production and preparation for food service (82).
- The United Tribes Technical College offers The Sustainable Agriculture and Food Systems Applied Science (AAS) degree program, which trains students in food production, nutrition, food preservation and storage, integrating tribal, cultural and traditional ecological knowledge (83).
- The University of Wisconsin-Madison Division of Extension Native Nations Team (84) and the College of Menominee Nation (85) are examples of institutions focusing on education to strengthen tribal food sovereignty and training in sustainable agriculture. They offer programs that engage youth in traditional foodways and support local economies through community food systems.
- The National Center for Home Food Preservation provides current science-based recommendations to process and preserve foods at home (2024).

## Conclusion and recommendations

Creating extension and outreach resources which summarize the applications of these preservation and processing technologies to indigenous commodity groups could make foods more stable, accessible, and safe, which support the food sovereignty. Mechanical drying technologies efficiently remove moisture and make foods stay longer; Thermal and nonthermal processes effectively reduce the microbial and chemical safety risks; Fermenting and pickling improve the nutritional value and flavor profile; Appropriate cold storage and packaging slow down the food spoilage and nutrition loss. Many of the abovementioned technologies are readily available at different scales inside and outside of indigenous communities. Despite these potential benefits and flexibility, several challenges remain for the wide adoption of them in nations.

Firstly, studies on processing and quality of North America indigenous foods are still limited in scientific research, which would provide the most direct information for indigenous people, and require interdisciplinary efforts from food science, engineering, nutrition, and health. Secondly, successful dissemination of food knowledge and resources relies on more effective and dedicated education, extension and outreach programs/initiatives. Finally, communication based on care and respect, and appreciation of the native food culture is critical for the alignment with the needs of the indigenous people and technology transfer. Addressing these will require universities/institutions, government agencies and private sectors to collaborate. While this type of collaboration may be a challenge, each agency is a stakeholder because enhancing the food sovereignty, nutrition security, and health equity in the underrepresented indigenous communities will contribute to a more sustainable global food system.

## Author contributions

DH: Data curation, Formal analysis, Investigation, Methodology, Visualization, Writing – original draft, Writing – review & editing. OP-Z: Formal analysis, Investigation, Methodology, Project administration, Resources, Supervision, Visualization, Writing – original draft, Writing – review & editing. CC: Conceptualization, Data curation, Formal analysis, Funding acquisition, Investigation, Methodology, Project administration, Resources, Supervision, Visualization, Writing – original draft, Writing – review & editing.
